# HAMLET Interacts with Lipid Membranes and Perturbs Their Structure and Integrity

**DOI:** 10.1371/journal.pone.0009384

**Published:** 2010-02-23

**Authors:** Ann-Kristin Mossberg, Maja Puchades, Øyvind Halskau, Anne Baumann, Ingela Lanekoff, Yinxia Chao, Aurora Martinez, Catharina Svanborg, Roger Karlsson

**Affiliations:** 1 Section of Microbiology, Immunology, and Glycobiology, Institute of Laboratory Medicine, Lund University, Lund, Sweden; 2 Department of Chemistry, University of Gothenburg, Göteborg, Sweden; 3 Department of Biomedicine, University of Bergen, Bergen, Norway; 4 Singapore Immunology Network, A*STAR, Singapore, Singapore; CNRS/Université de Toulouse, France

## Abstract

**Background:**

Cell membrane interactions rely on lipid bilayer constituents and molecules inserted within the membrane, including specific receptors. HAMLET (human α-lactalbumin made lethal to tumor cells) is a tumoricidal complex of partially unfolded α-lactalbumin (HLA) and oleic acid that is internalized by tumor cells, suggesting that interactions with the phospholipid bilayer and/or specific receptors may be essential for the tumoricidal effect. This study examined whether HAMLET interacts with artificial membranes and alters membrane structure.

**Methodology/Principal Findings:**

We show by surface plasmon resonance that HAMLET binds with high affinity to surface adherent, unilamellar vesicles of lipids with varying acyl chain composition and net charge. Fluorescence imaging revealed that HAMLET accumulates in membranes of vesicles and perturbs their structure, resulting in increased membrane fluidity. Furthermore, HAMLET disrupted membrane integrity at neutral pH and physiological conditions, as shown by fluorophore leakage experiments. These effects did not occur with either native HLA or a constitutively unfolded Cys-Ala HLA mutant (rHLA^all-Ala^). HAMLET also bound to plasma membrane vesicles formed from intact tumor cells, with accumulation in certain membrane areas, but the complex was not internalized by these vesicles or by the synthetic membrane vesicles.

**Conclusions/Significance:**

The results illustrate the difference in membrane affinity between the fatty acid bound and fatty acid free forms of partially unfolded HLA and suggest that HAMLET engages membranes by a mechanism requiring both the protein and the fatty acid. Furthermore, HAMLET binding alters the morphology of the membrane and compromises its integrity, suggesting that membrane perturbation could be an initial step in inducing cell death.

## Introduction

Lipid bilayers maintain cellular integrity by forming interactive barriers against the environment. They delineate the extracellular and intracellular spaces and play a critical role in the regulated uptake and export of essential components. Molecular interactions with this barrier *in vivo* may rely on lipid membrane constituents *per se*, or on specialized lipid-rich membrane domains, such as lipid rafts [1]. In addition, molecular recognition mechanisms often require specific receptors, which are embedded in lipid membranes, and structural modification of these receptors leads to the activation of highly regulated signaling pathways.

Membrane interactions are also essential in controlling cell survival and proliferation, and alterations in membrane composition and function accompany cancer development [2]. Molecules with selectivity for tumor cell membranes are important, to better understand the properties of tumor cells and to design agonists that attack and kill such cells with better selectivity. HAMLET (human alpha-lactalbumin made lethal to tumor cells) is a complex of partially unfolded α-lactalbumin (HLA) and oleic acid with broad tumoricidal activity. The complex interacts with tumor cell membranes, becoming rapidly internalized and translocated to the cell nuclei [3]. It appears that HAMLET triggers cell death by activating several signaling pathways and by interacting with cellular organelles, including proteasomes and mitochondria [4], [5]. HAMLET is also medically relevant, as demonstrated in clinical studies of human skin papillomas and bladder cancer [6], [7].

The mechanism of HAMLET uptake by tumor cells is unclear, however. HAMLET uptake has previously been quantified by radioactive labeling, quantitative confocal imaging and flow cytometry [4], [8]. Attempts have also been made to calculate the equivalents of membrane receptor sites. Such techniques are not fully applicable to HAMLET and tumor cells, however, due to rapid uptake of the complex and loss of membrane integrity as a result of the cell death process. Artificial membranes may thus be useful to specifically address how HAMLET interacts with lipid bilayers. The protein constituent of HAMLET, HLA, is a 14.2-kDa milk protein known to interact with different lipid membranes, with partially unfolded states like molten globules having increased affinity for lipid bilayers [9], [10], [11], [12]. Native, folded HLA alone is not efficiently internalized by tumor cells and has no effect on cell viability. Unfolding alone does not increase HLA uptake by tumor cells [13], [14], suggesting that there are major differences in how HAMLET and the fatty acid free protein component interact with a cell membrane, even after partial protein unfolding.

In this study, we examined the interactions of HAMLET with *in vitro* generated membranes of known composition, and compared HAMLET to the native or partially unfolded, fatty acid free proteins. We also examined the effect of HAMLET on plasma membrane vesicles (PMVs) obtained from tumor cells [15]. We show that HAMLET interacts with membranes and disturbs their integrity under physiological conditions. Binding to intact tumor cell membranes showed a patchy distribution, indicating that HAMLET may target molecules in specific membrane regions.

## Materials and Methods

### Materials

1,2-Dioleoylphosphatidylglycerol (DOPG), porcine brain phosphatidylserine (PBPS), soybean lecithin (polar extract) and egg yolk phosphatidylcholine (EYPC) lipids were from Avanti Polar Lipids (Alabaster, USA). Chemicals were from Merck (Darmstadt, Germany) and Sigma-Aldrich (St. Louis, MO, USA). 8-Aminonaphtalene-1,3,6-trisulfonic acid, disodium salt (ANTS), and p-xylene-bis-N-pyrimidinum bromide (DPX) used as a fluorophore and quencher, respectively, in the leakage assay were from Sigma-Aldrich. Chromatographic matrices were from Amersham Biosciences (Uppsala, Sweden) (HIC, DEAE-Sephacell) and DEAE-Trisacryl M from BioSephra (Villeneuf, France).

### HAMLET

The HAMLET complex was formed from partially unfolded HLA and oleic acid, as described [16]. Recombinant HLA^all-Ala^ was expressed in BL21* *Escherichia coli* using the T7-polymerase-based vector pAED4 (kindly donated by Peter S. Kim). Expression was induced by IPTG (Saveen &Werner AB; Limhamn, Sweden), rHLA^all-Ala^ was purified according to Kim *et al* [13] with slight modifications [14]. For fluorescence imaging, HAMLET, HLA and rHLA^all-Ala^, were labeled with AlexaFluor 568 (Molecular Probes, Carlsbad, CA, USA) according to the manufacture's instructions.

### Cellular Assays

A549 lung carcinoma (ATCC CCL-185) cells were cultured in RPMI with NEAA, sodium pyruvate and 5% fetal calf serum (FCS) and Pheochromocytoma (PC12) cells in RPMI with donor horse serum (10%) and FCS (5%) in collagen IV coated flasks (VWR International, USA). To study HAMLET uptake, A549 cells were grown overnight on poly-L-lysine chamber slides (Nunc, Thermofisher, Roskilde, Denmark) and treated with AlexaFluor 568-labeled HAMLET, native HLA or rHLA^all-Ala^ in medium without FCS (35 µM, 1 hour at 37°C in 5% CO_2_). Morphological changes and labeled protein uptake was recorded by real-time cellular imaging and confocal microscopy of fixed cells (LSM510 META confocal system, Carl Zeiss, Jena, Germany).

To quantify cell death, cells in RPMI without FCS were seeded into 24 well plates and incubated with different concentrations of each agonist at 37°C in 5% CO_2_. ATP measurements used ATPlite Luminescence ATP detection assay system (PerkinElmer, Waltham, MA, USA) and Trypan blue exclusion was recorded by interference contrast microscopy.

### Lipid Vesicles

Large Unilamellar Vesicles (LUVs) were produced from EYPC, PBPS and DOPG, as described [17], [18]. The prerequisite amounts of chloroform-dissolved lipids (1∶1 mixes of DOPG:EYPC and PBPS:EYPC, and EYPC alone) were transferred to Kimble glass tubes wrapped in aluminum foil. The chloroform solutions were dried to lipid films under dry N_2_-pressure, and residual chloroform was removed under vacuum. The samples were vortexed with 10 mM citric acid/20 mM Na_2_HPO_4_ buffer pH 7.0 and allowed to hydrate overnight. For liposome preparation, the solutions were subjected to seven freeze-thaw cycles using liquid N_2_ and a water bath at 55°C [19]. Hydrated multilamellar structures were extruded (LIPEX extruder, Northern Lipids, Burnaby, BC, Canada), forced through two Millipore filters (pore size 200 nm) 10 times using 12-bar N_2_ pressures and transferred to clean Kimble tubes for immediate usage. In the ANTS/DPX leakage experiments, the lipid film hydrating buffer contained 12.5 mM ANTS and 45 mM DPX. The free fluorophores and quenchers were removed by gel-filtration on a Sephadex G-75 exchange column.

A mix of multilamellar and unilamellar giant unilamellar vesicles (GUVs) were created from soybean lecithin (Polar extract, 45% phosphatidylcholine and 22% phosphatidylethanolamine; 18% phosphatidylinositol and 7% phosphatidic acid) or EYPC (72% phosphatidylcholine, 11% phosphatidylethanolamine and 16% other neutral lipids). Lipids were dissolved in chloroform (3 mg in 200 µl of chloroform), dried and then swelled in PBS, pH 7.2 (3 ml) overnight at 8°C and dispersed (1 mg lipid per ml) in an ultrasonic bath for 5 minutes. GUVs were then formed by the dehydration-rehydration method, which previously has been used for creating GUVs containing functional membranes with functional ion channels, used for patch-clamping [20]. The lipid film was allowed to swell on a glass bottom dish for microscopy (no. 1 glass thickness) for 5 minutes whereby surface-attached vesicles were formed.

### Surface Plasmon Resonance

The Biacore 3000 biosensor system (Biacore AB, Uppsala, Sweden) was used at 25°C, as described [21]. L1 sensor chip surfaces were cleaned with a 2-min injection of isopropanol: 50 mM NaOH (1∶1) at a flow rate of 20 µl/min, followed by 30 min washing with running buffer (HBS-N buffer: 10 mM HEPES, pH 7.4, 150 mM NaCl). LUV solutions were diluted with running buffer (pH 7.4) or acetate buffer (pH 5.0, 0.6–1 mM) and injected (flow rate of 10 µl/min), resulting in a deposition yielding measurements of 4000–6000 RU. The proteins (concentration range of 0–80 mM and dissolved in either buffer at pH 7.4 or pH 5.0, to match the buffer used in the previous step) were injected over the immobilized LUVs (flow rate of 100 µl/min). The sensor chip surface was regenerated by injecting isopropanol:50 mM NaOH (40∶60, v/v) and running buffer. Non-specific binding in the reference cell was subtracted. Binding affinity was estimated by fitting 3-parameter sigmoidal function to all concentration-RU data pairs, by nonlinear least-squares analysis, yielding S_0.5_-values reflecting the protein concentration necessary for a half-maximal response [21]. The fitting procedure also provided error estimates of the S_0.5_-values. The BIAevaluation program, version 3.2 (Biacore AS) was used for analysis of the sensorgrams. Curve fits were performed using Sigmaplot version 9.01 (Systat Software, Inc.).

### Fluorescence-Monitored Leakage Assays

LUV integrity was examined according to Ellens *et al* [22]. LUVs with ANTS/DPX encapsulation were diluted to 1 mM lipid concentration using citrate/Na_2_HPO_4_ at pH 5.0 or 7.0. Half-milliliter volumes were added to a 10 mm Hellma GmbH&Co quartz cuvette, and fluorescence was measured with a Perkin-Elmer LS5 Luminescence Spectrometer. Volumes of protein and oleic acid (OA) stock solution were added stepwise directly to the cuvette with gentle mixing (cumulative protein concentration 1, 5, 10, 20, 40, 60 and 80 mM). Fluorescence was recorded for each concentration (355 nm excitation, 450–550 nm emission, scan speed of 200 nm/min, 3 and 5 nm slit widths for the excitation and emission pathways), 1 min after addition of the protein 25°C. Spectra were dominated by ANTS fluorescence (λ_max_ at 510 nm). The emission intensity at this wavelength depends on ANTS/DPX proximity, and an increased intensity indicates loss of liposome integrity as the average distance between ANTS and DPX increases. Controls were measured and subtracted. Triton X-100 was added to the cuvette to a final concentration of 2 mM and fluorescence at 510 nm was arbitrarily set to 100%, i.e., complete LUV breakdown. Fluorescence at 510 nm prior to addition of stock solution was arbitrarily set to 0%, i.e., no leakage. This way of benchmarking the resulting change in fluorescence is convenient for comparing leakage responses for liposomes of a given lipid mix. The values represent the means of two experiments (<5% variation was obtained). The leakage assay is a well-established technique that has been optimized to make variations in quantum yield negligible [22], as recently reviewed and validated by Almeida and Pokorny [23]. Controls were performed to confirm that increases in fluorescence are not caused by changes in the molecular environment at the end-point of the experiments (i.e. by the presence of HAMLET, HLA and Triton X-100 at defined concentrations), and are uniquely associated to the leakage from liposomes. Briefly, controls consisted of measuring the effect of HAMLET or HLA addition, in the absence or presence of Triton X-100, on the fluorescence at 510 nm (355 nm excitation) of samples of ANTS and ANTS/DPX at different concentrations. The additions did not cause differences in the fluorescence intensity of ANTS.

### Imaging of HAMLET Interactions with Artificial Lipid Membranes

Alexa-labeled HAMLET or controls were added to GUVs in glass bottom dishes (0.5 mg/ml with 10% fluorescently labeled protein). Unbound protein was removed after 20 minutes using precision syringe pumping (15 minutes 2 ml/min). Microphotographs were taken before and after the labeled proteins were added, using confocal laser scanning microscopy (Leica TCS SP2 RS, Wetzlar, Germany), with a PL APO CS 63×/1.2 W corr objective for image acquisition. Brightness and contrast was adjusted in the Leica software. For intensity profile measurements, line scans were obtained with Image J software (http://rsbweb.nih.gov/ij/). An inverted fluorescence microscope (Leica DMI 6000B) was used to record morphological changes (bright-field, Leica DFC350FX camera system).

### Plasma Membrane Vesicles

Pheochromocytoma (PC12) cells were cultured for 2–4 days and plasma membrane vesicle (PMV) formation was induced as described by Bauer *et al* [15]. PC12 cells with surface-attached PMVs and free vesicles were then rinsed twice in washing buffer and transferred to a glass bottom dish (Wilco well) for incubation with Alexa-labeled proteins. PMVs from lung carcinoma cells (A549) were prepared by the same technique.

## Results

### 1. HAMLET Interacts with Neutral and Negatively Charged Membranes at Physiological pH

To understand the membrane affinity of HAMLET, we examined its interaction with biologically relevant membranes *in vitro*, using surface plasmon resonance with LUVs immobilized on a BiaCore L1 sensor chip.

**Figure 1:**
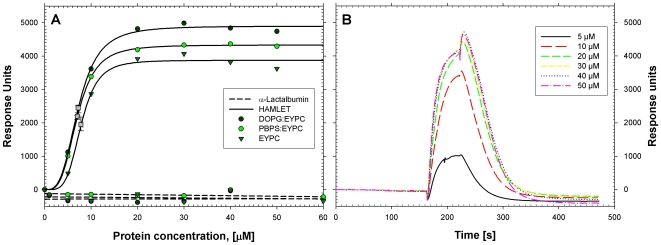
Interaction of HAMLET with vesicular membranes under physiological conditions. A) HAMLET and HLA interact with LUVs made of DOPG:EYPC (dark green cirkcle), PBPS:EYPC (light green circle) or EYPC (triangle) on BiaCore L1 sensor chips. Response Units were measured as a function of HAMLET (solid line) or HLA (dashed line) concentration. Three-parameter Hill-functions where fitted to the HAMLET data series, yielding S_0.5_-values, indicated by grey boxes in the figure for the interaction of the protein variant with LUVs made of DOPG:EYPC(S_0.5_ = 7.84±0.40), PBPS:EYPC (S_0.5_ = 6.99±0.07) or EYPC (S_0.5_ = 7.24±0.21) at pH 7.4. No HLA binding to the LUVs was detected under these conditions. B) Representative sensorgrams of HAMLET interacting with PBPS:EYPC at increasing concentrations of protein.

The composition of the LUVs chosen was based on previous studies of HLA interacting with and penetrating into such bilayers. Briefly, binding was optimal for lipid mixtures with an overall negative charge of 50% [24] achieved by 1∶1 mixtures containing either DOPG or PBPS as the negative component, and EYPC as the zwitterionic component. Liposomes prepared with DOPG or PBPS alone would contribute 100% negative charge, while EYPC-only liposomes would have 0%; thus, a 1∶1 mixture yields 50% negative charge. As fluidity also defines the membrane quality of potential importance for HLA-membrane interactions{Agasoster, 2003 #165},three types of LUVs (DOPG:EYPC, PBPS:EYPC and EYPC lipid mixtures) representing different states of fluidity and charge were used to investigate HAMLET binding (Fig. 1A). The DOPG:EYPC liposomes are in the fluid l_d_-state, while the two latter species are close to their transition temperature, T_c_ [24], [25]. Since 18∶1 oleic acid acts as the HAMLET co-factor, the amount of 18∶1 fatty acid 18∶1 acyl chains in the liposomes is of interest when interpreting the binding results. The DOPG:EYPC lipid mix contains 66% 18∶1 acyl chains, while the PBPS:EYPC and EYPC mixtures contain approximately 33%.

HAMLET bound strongly and in a concentration-dependent manner to all three membranes at near physiological conditions (pH 7.4, 150 mM NaCl), with only small differences in absolute binding and S_0.5_ values (S_0.5_-values ranging from 6.99–7.84, with standard errors in the range of 0.07–0.40; Figs. 1A and B). HAMLET binding was not influenced by membrane fluidity, charge or 18∶1 fatty acid acyl chain composition. HLA did not bind to any of the LUVs under physiological conditions (Fig. 1A), confirming previous studies where a pH<5 was optimal for HLA binding to bilayers with 50% negatively charged phospholipids. The results show that HAMLET binds broadly to membranes under physiological conditions, in contrast to HLA.

### 2. HAMLET Interacts with Giant Unilamellar Vesicle Membranes

The membrane interactions of HAMLET were further investigated by confocal microscopy imaging of GUVs composed of EYPC or soybean lipids at pH 7. LUVs were not suitable for confocal imaging, as they do not form stable unilamellar vesicles under these conditions. The GUV's, prepared by dehydration-rehydration, yielded a mixture of unilamellar (single layer) and multilamellar (several layers) phospholipid membranes. The membrane properties of the respective vesicles were the same, however, regardless of size and structure. Conclusions were based on pair-wise analysis within each preparation.

**Figure 2:**
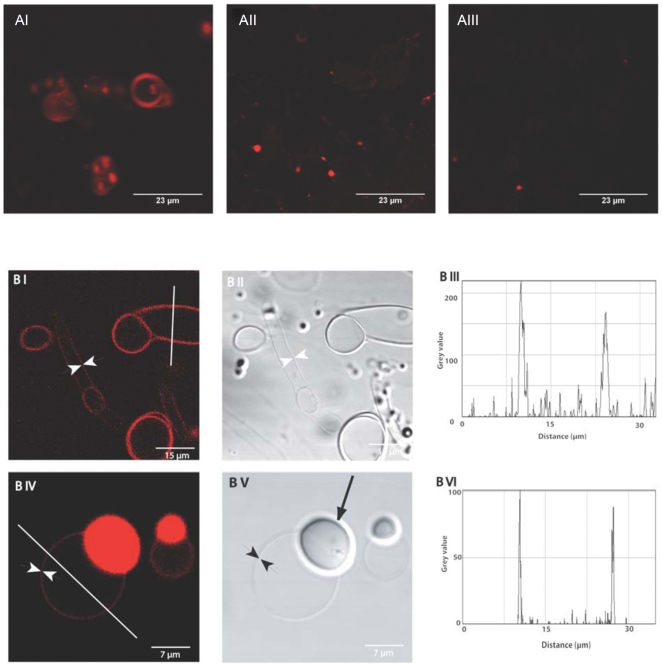
Confocal imaging of HAMLET binding to lipid membranes in GUVs. A. Differential interference contrast images and corresponding fluorescence confocal microscopy images. EYPC lipid vesicle membranes were visualized after application of Alexa-labeled HAMLET (I), HLA (II) or free Alexa 568 dye (III). HAMLET bound to multiple layers of lipid membranes in the multilamellar vesicles. Vesicles after application of Alexa-labeled HLA or free Alexa 568 dye indicated weak fluorescence association. B. Membrane detection of LUVs by fluorescence confocal microscopy, and corresponding intensity profile measurements, using ImageJ software and a median filter radius of 1.0 pixels to process the images before the profile measurements. I & II) Binding of HAMLET to EYPC vesicles; confocal micrograph and corresponding DIC microscopy images. White arrowheads illustrate unilamellar structures. III) Intensity profile measurement across the line shown in I, confirming the strong association of HAMLET to the membrane. IV) Binding of Alexa-labeled HAMLET to soybean phorphoryl choline vesicles. V) Corresponding DIC micrograph. Black arrowheads illustrate unilamellar structures and the black arrow indicates a multilamellar structure. VI) Intensity profile measurement of the line shown in IV. HAMLET bound strongly to soybean vesicle membranes.

Membranes of EYPC lipid vesicles were clearly visualized by binding of fluorescently labelled HAMLET, whereas HLA and free Alexa 568 dye associated more weakly to the vesicles, as shown by the low fluorescence signal (Fig. 2A). To quantify the membrane association of HAMLET in unilamellar vesicles, we used intensity profile measurements with ImageJ software and a median filter radius of 1.0 pixels. There was a clear HAMLET association with both EYPC and soybean membranes, but only a weak signal inside the unilamellar vesicle, suggesting no substantial internalization of the HAMLET complex (Fig. 2B).

The results show that HAMLET binds more strongly than native HLA to both neutral and slightly negatively charged artificial membranes.

### 3. Membrane Perturbations by HAMLET

**Figure 3:**
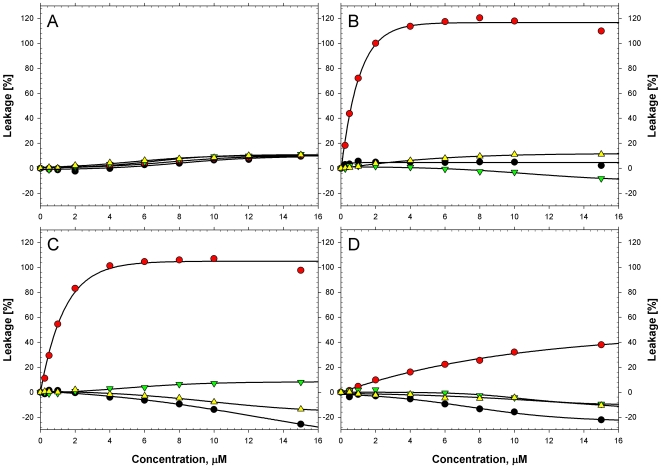
Leakage of ANTS/DPX from LUVs in response to HAMLET, monitored by fluorescence. ANTS/DPX fluorophore leakage across liposome membranes after one-minute exposure to HAMLET (red circle), HLA (black circle), water (yellow triangle) or oleic acid (green triangle). A) EYPC LUVs at pH 7.0. B) DOPG:EYPC liposomes at pH 7.0. C) PBPS:EYPC at pH 7.0. D) EYPC LUVs at pH 5.0. The fluorescence (at 510 nm) of undisturbed LUVs was set to 0% and that of vesicles disrupted by Triton X-100 was set to 100%. The leakage response is presented as a function of polypeptide/oleic acid concentration in the fluorescence cuvette, except for water, which was added to reach the intended concentration of polypeptide/oleic acid.

To test if membrane integrity was affected by HAMLET, LUVs were loaded with ANTS fluorophore and DPX quencher, and leakage into the extravesicular space was quantified by measuring ANTS fluorescence. Native α-lactalbumin disrupts liposomes at pH 5, but has no effect at neutral pH or on zwitterionic liposomes at acidic pHs [24]. HAMLET disrupted negatively charged (DOPG:EYPC and PBPS:EYPC) after one minute at neutral pH (Fig. 3B,C). Fluorophore leakage was higher for DOPG:EYPC, followed by PBPS:EYPC (Fig. 3B and C), indicating that the degree to which HAMLET can alter membrane structure is influenced by the membrane's lipid composition and physical characteristics. HAMLET caused a maximal leakage, 100–120% relative to that induced by the Triton X-100 control, used for benchmarking the leakage response within each experimental series on a given liposome lipid mix. Such high relative permeability suggests that HAMLET is a very effective disruptor of negatively charged membranes at neutral pH. HAMLET bound zwitterionic vesicles (EYPC) (Fig. 1A) and although no disruption was observed at neutral conditions (Fig. 3A), leakage was measured at pH 5.0 (Fig. 3D). Free oleic acid had no detectable effect on LUVs independent of phospholipidic composition (Fig. 3). Thus, HAMLET, but not HLA or oleic acid, permeabilizes negatively charged lipid bilayers under physiological conditions. Moreover, HAMLET is a very efficient disruptor of DOPG:EYPC and PBPS:EYPC vesicles, with the former, enriched in oleoyl-sidechains, being even more susceptible to disruption by HAMLET than by Triton X-100.

**Figure 4:**
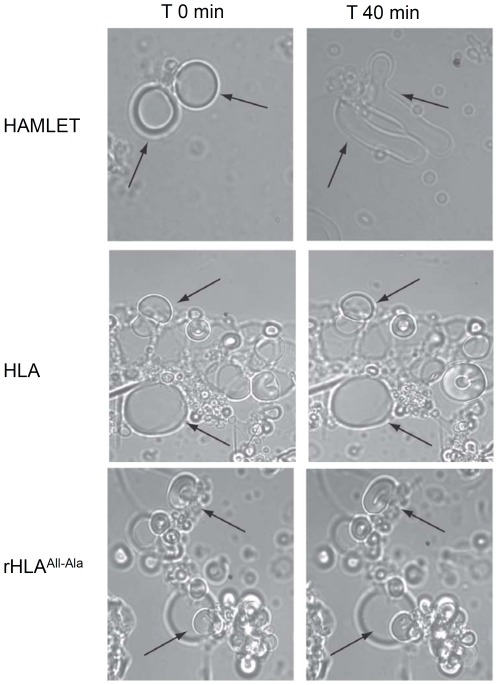
HAMLET alters the morphology of lipid vesicles. Bright-field micrographs showing glass-adherent egg yolk vesicles before and after a 40-minute exposure to HAMLET, HLA or rHLA^all-Ala^. At T = 0, the vesicles were rigid, with predominantly rounded morphology. Following HAMLET exposure, unilamellar structures were more prominent, the membranes were elongated and an increase in thermal mobility and fluidity of the membranes was observed (see arrows, upper panels). The morphology of vesicles treated with HLA or rHLA^all-Ala^ was unchanged (see arrows, lower panels).

We also documented the effect of HAMLET on membranes by light microscopy. Vesicles made of EYPC in PBS buffer at pH 7 remained morphologically unchanged for one hour, but the addition of HAMLET affected the shape of both multi- and unilamellar vesicles. We observed a change from round and defined to elongated, heterogeneous morphology after 40 minutes (Fig. 4). In addition, continuous monitoring revealed an increase in membrane movement, with a subsequent increase in the number of unilamellar structures (data not shown). The contribution of protein unfolding to this change in membrane morphology was examined by comparing HAMLET to native HLA and to the rHLA^all-Ala^ mutant, which lacks disulphide bonds and is therefore unable to fold into the native state. Morphological changes of membrane vesicles were not observed after treatment with either native HLA or rHLA^all-Ala^.

The results show that HAMLET, but not HLA or rHLA^all-Ala^, alters the morphology of membrane vesicles.

### 4. Interaction of HAMLET with PMVs from Intact Tumor Cells

To compare the effect of HAMLET on artificial protein-free membranes versus tumor cell membranes (containing membrane receptors), we prepared PMVs from the tumor cells PC12 [15]. Free PMVs and PMVs (blebs) still attached to cells were exposed to Alexa-labeled HAMLET at concentrations corresponding to the LD_50_ cell death value (0.5 mg/ml). Alexa-labeled HLA and free Alexa dye were used as controls.

**Figure 5:**
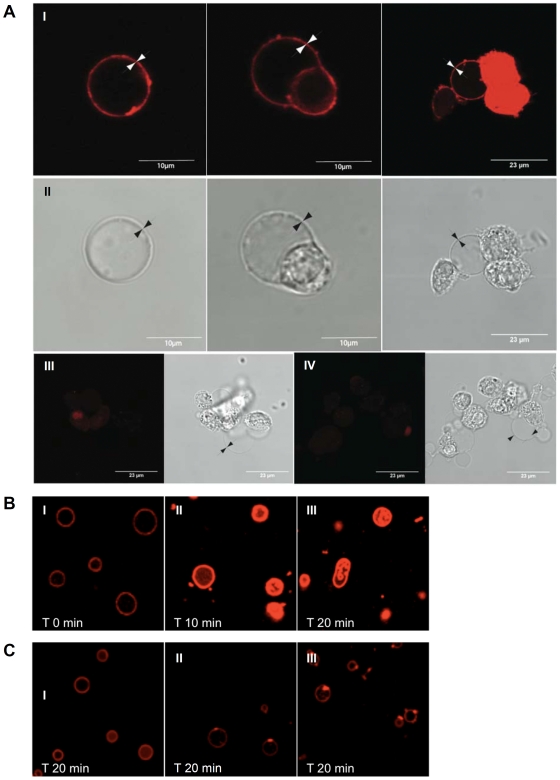
Interaction of HAMLET with tumor cell PMVs. Pheochromocytoma cell (PC12) PMVs on glass-bottom dishes were exposed to Alexa-labeled HAMLET, Alexa-labeled HLA or free Alexa 568 dye. Membrane fluorescence was observed by confocal fluorescence microscopy and morphology by differential interference contrast microscopy. A) Detection of Alexa-HAMLET on PMVs (I, white arrowheads). Cell remnants are also highly stained (I, right panel). Black arrowheads indicate the PMV membrane (II). Weak detection of PMVs and cell remnants after application of Alexa-labeled HLA (III). Free Alexa dye served as a negative control (IV). B) PMVs from A549 cells exposed to HAMLET (35 µM, 0, 10, 20 min) and visualized with Nile red. An increase in fluorescence intensity and change of shape was observed over time. Internal convoluted membrane structures developed (arrows). C) PMVs from A549 cells exposed to OA and visualized with Nile red (0, 10, 20 min) showed a gradual increase in membrane binding at distinct membrane spots (arrows).

HAMLET bound strongly to tumor cell PMVs (Fig. 5A, I & II). In contrast to the artificial protein-free membranes, the HAMLET distribution on PMVs was patchy, indicating that specific membrane regions may have higher affinity for the complex. There was no clear evidence of translocation across the membrane into the interior of the PMVs, but the remainder of the tumor cells from which the blebs had been derived fluoresced strongly, showing that Alexa-labeled HAMLET had been taken up. Alexa-labeled HLA bound weakly to the PMVs and cell remnants (Fig. 5A, III). Alexa dye did not interact with the PMVs *per se*, but cells fluoresced weakly, possibly due to non-specific uptake through the ruptured plasma membrane (Fig. 5A, IV).

To confirm our results, we tested in parallel PMVs from another tumor cell line, A549 lung carcinoma cells, and used a different visualization marker. The vesicles also bound HAMLET with a patchy distribution, as visualized with Nile red detection (Fig. 5B I–III). After 20 minutes, a change in membrane morphology was observed, with an accumulation of Nile red in the membranes accompanied by elongated, convoluted shapes.

### Oleic Acid Binds PMVs but Does Not Disrupt LUVs or GUVs

In parallel with HAMLET and HLA, the different vesicle preparations (LUVs, GUVs, and PMVs) were exposed to OA, at a concentration corresponding to the stoichiometry of 4–6 OA residues per HLA in the HAMLET complex. In ANTS/DPX-loaded vesicles, OA alone did not cause an increase in extravesicular fluorescence, indicating that the membranes remained intact (Fig. 3). In the membrane morphology studies, OA alone did not cause any detectable change in vesicle shape or flexibility (data not shown). OA did incorporate into PMV membranes. However, OA became associated with the PMVs, resulting in weak fluorescence compared to HAMLET, but concentrated in spots on the membrane (arrows in Fig. 5C I–III) indicating an accumulation in membrane dots. There was no change in PMV morphology comparable to that seen with HAMLET.

### HAMLET Internalization and Cell Death Require Both Unfolded HLA and Oleic Acid

**Figure 6:**
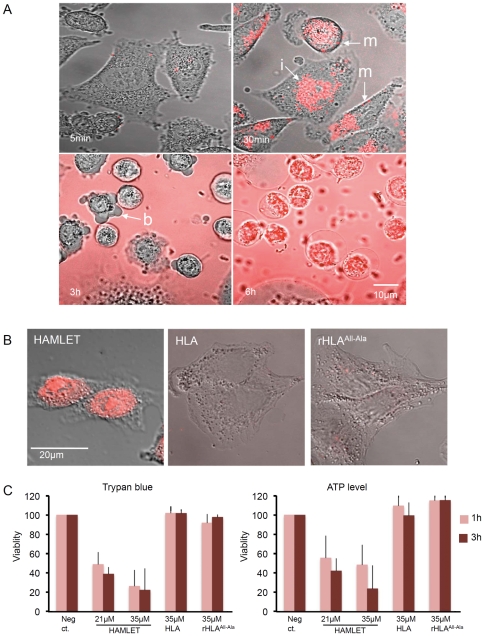
Membrane interactions of HAMLET and HLA variants, uptake and tumor cell death. A) Progressive Alexa-HAMLET (21 µM) internalization by tumor cells from 30 minutes to six hours. Arrows indicate membrane (m), internalization (i) and blebs (b). B) Fixed A549 cells showing Alexa-HAMLET internalization into the cytoplasm and nuclei (one hour). The oleic acid free proteins Alexa-HLA and Alexa-rHLA^all-Ala^ were not significantly internalized (three hours). C) Loss of cell viability, quantified by trypan blue exclusion and ATP measurement. Cells started to die after one hour of incubation with 21 or 35 µM of HAMLET, but not when exposed to native HLA or rHLA^all-Ala^.

To further examine membrane binding of intact tumor cells, A549 lung carcinoma cells were exposed to Alexa-labeled HAMLET or HLA. Membrane interactions and effects on tumor cell morphology were first recorded by live-cell imaging (Fig. 6A). HAMLET rapidly became associated with the cell membrane, and internalization occurred immediately (Fig. 6A).

Over a six-hour period, HAMLET-treated cells changed morphologically. HAMLET became membrane-associated and internalized after 30 minutes, and after three hours the cells rounded up and formed blebs. After six hours, membrane integrity was lost and all cells had internalized HAMLET (Fig. 6A). There was no binding, uptake or morphological change after cell exposure to the protein component of HAMLET alone. Neither fatty acid free HLA nor the unfolded mutant, rHLA^all-Ala^, entered or killed cells (Fig. 6B). In HAMLET-treated cells, tumor cell death coincided with HAMLET internalization, and was detected by trypan blue exclusion and ATP measurements after one- and three-hour exposures, (Fig. 6C).

The results show that membrane binding and internalization by tumor cells require partially unfolded HLA and oleic acid. Importantly, unfolding alone did not enable HLA to bind to cell membranes, become internalized or kill carcinoma cells.

## Discussion

The tumoricidal HAMLET complex interacts with the tumor cell membrane, is internalized and translocates to the cell nucleus [3]. This study used artificial membranes to define whether HAMLET can interact with phospholipid bilayers in the absence of specific tumor cell membrane constituents. We showed that HAMLET bound membranes at physiological pH, and as a result, perturbed the membrane integrity and caused leakage of vesicular contents to the exterior. In addition, the morphology of vesicles composed of natural lipid mixtures was altered. The effects were quite dramatic, differing markedly from those of natively folded HLA, which failed to bind membranes at neutral pH. Significant HLA binding has only been recorded at pH<5, where the proton gradient near the negatively charged membrane is thought to be sufficient to remove negative charges at key residues, and thus drive protein unfolding necessary for membrane binding [11], [26], [27]. To determine whether protein unfolding is sufficient to create a membrane-interactive form of HLA, we used the mutant rHLA^all-Ala^, which lacks all four disulfide bonds and therefore cannot adopt the native HLA fold [14]. This mutant did not bind to membranes to the same extent as HAMLET or perturb membrane function at conditions amenable for HAMLET binding. The results suggest that protein unfolding and the fatty acid together increase membrane affinity and that the oleic acid bound form engages membranes by a mechanism requiring both the protein and the fatty acid.

The artificial, protein-free vesicles in this study contained phospholipid extracts from natural sources (egg yolk, porcine brain and soybean) and one synthetic lipid (DOPG) carrying two oleic acids. The extracts were used to ensure that the fluidity and charge of the membranes reflected naturally occurring compositions [28]. The synthetic lipid was added to test the effect of oleic acid moieties on the membrane. HAMLET bound to the artificial membranes regardless of lipid composition, charge or oleic acyl chain content. In contrast to HLA and derivatives, HAMLET even bound to membranes made only of EYPC, which has an overall neutral charge under these experimental conditions, indicating that the interaction is not dominated by long-range columbic interactions. For native HLA, at least, membrane curvature is of secondary importance for binding relative to membrane charge and the protein's membrane-bound folding state [24], [28], [29]. That HAMLET has high affinity for a range of liposomes examined here (neutral, charged, LUVs and GUVs) suggests that curvature is also less important for the partially folded protein. A previous study [25] of SUVs prepared with similar lipid mixtures suggested that at room temperature the mixtures are either in the l_d_-state (DOPG:EYPC) or within the transition temperature interval (EYPC, PBPS:EYPC). Moreover, HLA increases the membrane T_c_ upon binding. Whether this latter feature is true of HAMLET is an open question, but we interpreted this as indicating that the degree to which the native protein penetrates the membrane and the physical state of the membrane were coupled.

Thermodynamic access to intermediately folded protein states has been proposed to drive membrane binding of proteins, including HLA [27], [30], [31], [32]. Membrane curvature is a known contributor in many systems. However, for HLA interacting with negatively charged membranes, this factor is less important than bulk conditions and membrane charge [24], [28]. For HAMLET, unfolding of HLA alone was not sufficient to create membrane affinity and to permeabilize the membrane, emphasizing that the role of oleic acid, in addition to kinetically trapping HAMLET in its active conformation, might be to enhance membrane affinity. Increased hydrophobicity through the fatty acid acyl-chain, or modulation of the local charges on the protein through shielding and hydrogen bonding are possibilities. It remains unclear if short-range forces could consolidate the interaction with the subset of conformations available that are most favorable for binding the membrane.

Free fatty acids are used by all cells as an energy source and as building blocks for hormones, glycolipids and lipoproteins and are constantly taken up by cells. Although the uptake mechanisms are not fully understood, a range of receptors and transport systems are thought to be involved [33]. Fatty acids also cross lipid bilayers by receptor-independent mechanisms; this has been ascribed to their hydrophobicity [34]. We have confirmed elsewhere that tumor cells internalize free oleic acid as well as the HAMLET complex, suggesting that the primary membrane interactions may depend on oleic acid as well as the protein (manuscript in preparation). In the present study, neither HLA nor oleic acid alone could alter membrane structure or disrupt vesicles, suggesting that both the unfolded protein and the lipid are necessary bind to and disrupt membranes.

The difference in membrane affinity between HAMLET and HLA was confirmed using tumor cell PMVs, suggesting that the artificial membrane systems might reflect important properties of intact cell membranes. Previous studies using microscopy and proteomic analysis of cytosolic proteins have shown that the PMV interior is filled with cytosolic molecules but does not contain cytoskeletal elements such as actin or microtubules [35]. The lipid composition of the PMVs is representative of the donor cell plasma membrane, with phospholipid/cholesterol ratios of 2∶1 [36] and PMVs display phase separation of proteins and lipids [37]. HAMLET was distributed in patches on PMV membranes, indicating that specific membrane regions bound HAMLET more strongly than others. Thus, whereas lipid bilayers and their constituents are sufficient for HAMLET binding, specific receptors for HAMLET or oleic acid could also contribute in tumor cell interaction. This is consistent with preliminary studies indicating that HAMLET internalization is influenced by specific signaling pathways and by the metabolic state of tumor cells (P. Storm *et al* manuscript in preparation). The membrane interactions described here may allow the complex to reach specific membrane-associated receptor structures, thus enabling HAMLET to activate the complex signaling pathways involved in cell death.

## References

[1] Lajoie P, Goetz JG, Dennis JW, Nabi IR (2009). Lattices, rafts, and scaffolds: domain regulation of receptor signaling at the plasma membrane.. J Cell Biol.

[2] Baritaki S, Apostolakis S, Kanellou P, Dimanche-Boitrel MT, Spandidos DA (2007). Reversal of tumor resistance to apoptotic stimuli by alteration of membrane fluidity: therapeutic implications.. Adv Cancer Res.

[3] Duringer C, Hamiche A, Gustafsson L, Kimura H, Svanborg C (2003). HAMLET interacts with histones and chromatin in tumor cell nuclei.. J Biol Chem.

[4] Gustafsson L, Aits S, Onnerfjord P, Trulsson M, Storm P (2009). Changes in proteasome structure and function caused by HAMLET in tumor cells.. PLoS One.

[5] Kohler C, Gogvadze V, Hakansson A, Svanborg C, Orrenius S (2001). A folding variant of human alpha-lactalbumin induces mitochondrial permeability transition in isolated mitochondria.. Eur J Biochem.

[6] Gustafsson L, Leijonhufvud I, Aronsson A, Mossberg AK, Svanborg C (2004). Treatment of skin papillomas with topical alpha-lactalbumin-oleic acid.. N Engl J Med.

[7] Mossberg AK, Wullt B, Gustafsson L, Mansson W, Ljunggren E (2007). Bladder cancers respond to intravesical instillation of HAMLET (human alpha-lactalbumin made lethal to tumor cells).. Int J Cancer.

[8] Hakansson A, Zhivotovsky B, Orrenius S, Sabharwal H, Svanborg C (1995). Apoptosis induced by a human milk protein.. Proc Natl Acad Sci U S A.

[9] Banuelos S, Muga A (1995). Binding of molten globule-like conformations to lipid bilayers. Structure of native and partially folded alpha-lactalbumin bound to model membranes.. J Biol Chem.

[10] Banuelos S, Muga A (1996). Structural requirements for the association of native and partially folded conformations of alpha-lactalbumin with model membranes.. Biochemistry.

[11] Halskau Ø, Frøystein NA, Muga A, Martinez A (2002). The membrane-bound conformation of alpha-lactalbumin studied by NMR-monitored 1H exchange.. J Mol Biol.

[12] Zherelova OM, Kataev AA, Grishchenko VM, Knyazeva EL, Permyakov SE (2009). Interaction of antitumor alpha-lactalbumin-oleic acid complexes with artificial and natural membranes.. J Bioenerg Biomembr.

[13] Peng ZY, Wu LC, Kim PS (1995). Local structural preferences in the alpha-lactalbumin molten globule.. Biochemistry.

[14] Pettersson-Kastberg J, Mossberg A, Trulsson M, Yong JY, Soyung M (2009). a-Lactalbumin, engineered to be non-native and inactive, kills tumor cells when in complex with oleic acid.. Journal of Molecular Biology, in press.

[15] Bauer B, Davidson M, Orwar O (2006). Direct reconstitution of plasma membrane lipids and proteins in nanotube-vesicle networks.. Langmuir.

[16] Svensson M, Hakansson A, Mossberg AK, Linse S, Svanborg C (2000). Conversion of alpha-lactalbumin to a protein inducing apoptosis.. Proc Natl Acad Sci U S A.

[17] Burgess SW, McIntosh TJ, Lentz BR (1992). Modulation of poly(ethylene glycol)-induced fusion by membrane hydration: importance of interbilayer separation.. Biochemistry.

[18] Mayer LD, Bally MB, Hope MJ, Cullis PR (1986). Techniques for encapsulating bioactive agents into liposomes.. Chem Phys Lipids.

[19] Mayer LD, Hope MJ, Cullis PR, Janoff AS (1985). Solute distributions and trapping efficiencies observed in freeze-thawed multilamellar vesicles.. Biochim Biophys Acta.

[20] Criado M, Keller BU (1987). A membrane fusion strategy for single-channel recordings of membranes usually non-accessible to patch-clamp pipette electrodes.. FEBS Lett.

[21] Cho W, Bittova L, Stahelin RV (2001). Membrane binding assays for peripheral proteins.. Anal Biochem.

[22] Ellens H, Bentz J, Szoka FC (1984). pH-induced destabilization of phosphatidylethanolamine-containing liposomes: role of bilayer contact.. Biochemistry.

[23] Almeida PF, Pokorny A (2009). Mechanisms of antimicrobial, cytolytic, and cell-penetrating peptides: from kinetics to thermodynamics.. Biochemistry.

[24] Rodland I, Halskau O, Martinez A, Holmsen H (2005). alpha-Lactalbumin binding and membrane integrity–effect of charge and degree of unsaturation of glycerophospholipids.. Biochim Biophys Acta.

[25] Agasoster AV, Halskau O, Fuglebakk E, Froystein NA, Muga A (2003). The interaction of peripheral proteins and membranes studied with alpha-lactalbumin and phospholipid bilayers of various compositions.. J Biol Chem.

[26] Johnson JE, Xie M, Singh LM, Edge R, Cornell RB (2003). Both acidic and basic amino acids in an amphitropic enzyme, CTP:phosphocholine cytidylyltransferase, dictate its selectivity for anionic membranes.. J Biol Chem.

[27] Halskau Ø, Muga A, Martinez A (2009). Linking new paradigms in protein chemistry to reversible membrane-protein interactions.. Curr Protein Pept Sci.

[28] Halskau O, Ying M, Baumann A, Kleppe R, Rodriguez-Larrea D (2009). Three-way interaction between 14-3-3 proteins, the N-terminal region of tyrosine hydroxylase, and negatively charged membranes.. J Biol Chem.

[29] Halskau O, Froystein NA, Muga A, Martinez A (2002). The membrane-bound conformation of alpha-lactalbumin studied by NMR-monitored 1H exchange.. J Mol Biol.

[30] Halskau Ø, Underhaug J, Frøystein NA, Martinez A (2005). Conformational flexibility of alpha-lactalbumin related to its membrane binding capacity.. J Mol Biol.

[31] Halskau Ø, Perez-Jimenez R, Ibarra-Molero B, Underhaug J, Munoz V (2008). Large-scale modulation of thermodynamic protein folding barriers linked to electrostatics.. Proc Natl Acad Sci U S A.

[32] van der Goot FG, Gonzalez-Manas JM, Lakey JH, Pattus F (1991). A ‘molten-globule’ membrane-insertion intermediate of the pore-forming domain of colicin A.. Nature.

[33] Hamilton JA (1998). Fatty acid transport: difficult or easy?. J Lipid Res.

[34] Kleinfeld AM (2000). Lipid phase fatty acid flip-flop, is it fast enough for cellular transport?. J Membr Biol.

[35] Bauer B, Davidson M, Orwar O (2009). Proteomic analysis of plasma membrane vesicles.. Angew Chem Int Ed Engl.

[36] Fridriksson EK, Shipkova PA, Sheets ED, Holowka D, Baird B (1999). Quantitative analysis of phospholipids in functionally important membrane domains from RBL-2H3 mast cells using tandem high-resolution mass spectrometry.. Biochemistry.

[37] Baumgart T, Hammond AT, Sengupta P, Hess ST, Holowka DA (2007). Large-scale fluid/fluid phase separation of proteins and lipids in giant plasma membrane vesicles.. Proc Natl Acad Sci U S A.

